# Biological Aging and Immune Senescence in Children with Perinatally Acquired HIV

**DOI:** 10.1155/2020/8041616

**Published:** 2020-05-16

**Authors:** Annalisa Dalzini, Maria Raffaella Petrara, Giovanni Ballin, Marisa Zanchetta, Carlo Giaquinto, Anita De Rossi

**Affiliations:** ^1^Section of Oncology and Immunology, Department of Surgery, Oncology and Gastroenterology, Unit of Viral Oncology and AIDS Reference Center, University of Padova, Padova, Italy; ^2^Veneto Institute of Oncology IOV – IRCCS, Padua, Italy; ^3^Department of Mother and Child Health, University of Padova, Padova, Italy

## Abstract

Chronic HIV-infected children suffer from premature aging and aging-related diseases. Viral replication induces an ongoing inflammation process, with the release of pathogen-associated molecular patterns (PAMPs) and damage-associated molecular patterns (DAMPs), the activation of the immune system, and the production of proinflammatory cytokines. Although combined highly active antiretroviral therapy (ART) has significantly modified the natural course of HIV infection, normalization of T and B cell phenotype is not completely achievable; thus, many HIV-infected children display several phenotypical alterations, including higher percentages of activated cells, that favor an accelerated telomere attrition, and higher percentages of exhausted and senescent cells. All these features ultimately lead to the clinical manifestations related to premature aging and comorbidities typically observed in older general population, including non-AIDS-related malignancies. Therefore, even under effective treatment, the premature aging process of HIV-infected children negatively impacts their quality and length of life. This review examines the available data on the impact of HIV and ART on immune and biological senescence of HIV-infected children.

## 1. Introduction

The natural history of HIV infection has greatly changed over the course of the last 20 years due the great improvement of the combined highly active antiretroviral therapy (ART). Compared to the past, life expectancy of HIV-infected individuals on ART has drastically increased; however, ART does not eradicate the infection; therefore, HIV will persist in infected individuals, becoming a chronic disease [1]. Although some studies suggested that, under optimal treatment, life expectancy could be similar to that of the uninfected population [2–4], other studies evidenced that this goal has not been achieved yet, and life expectancy in Western countries can be shortened of up to 10 years [5–8]. AIDS-related complications, among which opportunistic infections and AIDS-defining malignancies, are reduced compared to the past; however, HIV-infected individuals on ART still have a higher risk of non-AIDS-related morbidity and mortality, due to an increased incidence of a wide range of illnesses associated with aging [9, 10].

Aging is a natural process that involves the loss of physiological integrity with a generalized organ decline that ultimately leads to death [11]; an aging system faces a decreasing ability to deal with stress and increasing frailty [12–15], inflammation [12], and age-related comorbidities, including cardiovascular disease, neuropathy, anemia, osteoporosis, and liver and kidney disease [11, 16]. The persistence of HIV, causing chronic immune activation, is likely a key determinant of the premature senescent pathway. Indeed, viral persistence induces activation of immune system cells, which undergo continuous expansion as a response to the antigen, eventually reaching the senescent stage, when they lose their functions [17]. A direct consequence of cellular replication is the shortening of their telomeres, until they reach a critical length under which the replicative capacity of the cell is lost [18–20], fueling the cells' premature senescence and the development of those age-related diseases that are involved the loss of the regenerative capacity of different tissues [21]. It is nowadays well established that there is a link between telomere shortening, cellular senescence, and aging [22]. In addition, HIV itself can impair the activity of telomerase (a ribonucleoprotein enzyme complex that synthesizes the telomeric repeats TTAGGG [23, 24]) specifically in CD4 cells [25]. The importance of this adverse effect resides on the fact that although telomerase is usually not expressed in somatic cells, it can be transiently upregulated in lymphocytes upon cell activation [26, 27]; the impairment of this upregulation can therefore increase the apoptotic propensity of hematologic cells and lead to immune system dysfunction.

Currently about 38 million people are living with HIV; 2 million of them are children under 15 years of age. Although new HIV infections among children are steadily decreasing, still, 160000 new infections occurred in 2018, the vast majority of them being mother-to-child transmission (MTCT) in African countries [28]. The clinical complications of HIV infection in children are more serious than those in adults [29–32]. Indeed, they experience a poorer control of the disease, which progresses to AIDS faster [29, 33], and the acute stage of the infection is characterized by higher levels of viremia, which is controlled slower and less effectively than in adults [33–35]. Several studies conducted in children [36–39] suggest that possible causes of the differences mentioned above include the very early exposure to HIV and pathogens when the infants' immune system is still under development, an interaction that could also influence the evolution of their incomplete immune system.

Thanks to the continuously increasing coverage of ART-based prophylaxis and treatment, in 2018, MTCT incidence was under 2%, and about 50% of HIV-infected children were receiving ART [28]. Therefore, an increasing number of children start ART at a very young age and will be receiving antiretroviral drugs for all their lifetime. ART greatly improved their survival and the quality of life [40, 41], but on the other hand, they now face the consequences of a lifelong chronic condition, suffering from pathogenic mechanisms typical of premature aging [42–46]; i.e., they show an increased risk of age-associated comorbidities, identified as non-AIDS-related diseases [47–49], compared to healthy individuals [50–52]. It has been suggested that antiretroviral drugs themselves can impact on accelerated aging, mainly due to the inhibitory effect of nucleoside reverse transcriptase inhibitors (NRTIs) on telomerase [53, 54]; however, more recent studies argued that the effect of prophylaxis and therapy is negligible compared to that of the HIV infection itself [55, 56].

**Figure 1:**
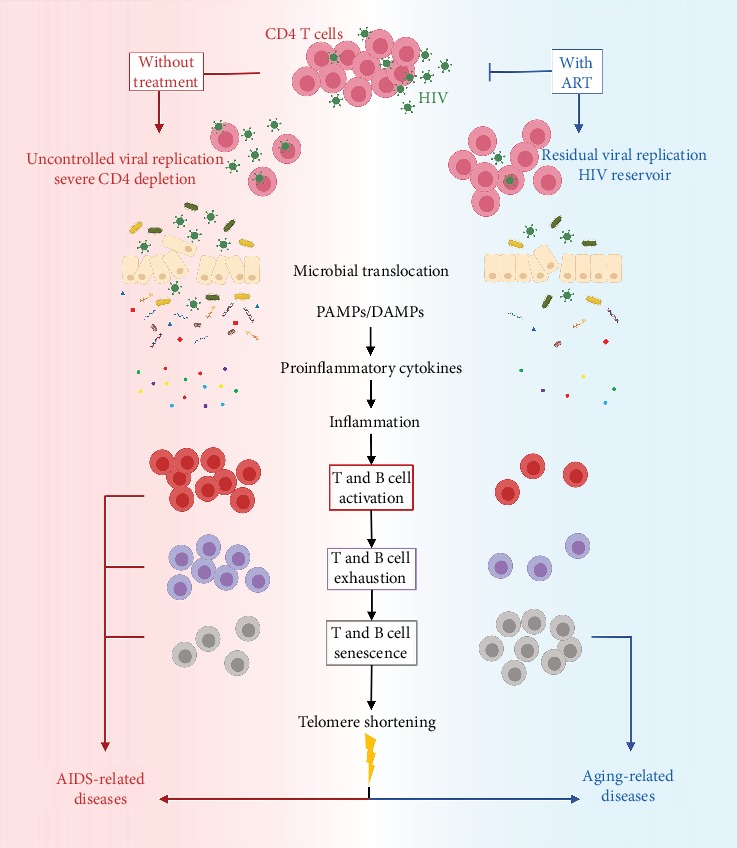
Schematic representation of the impact of HIV without (left side, red) and with (right side, blue) ART. HIV infects primarily CD4 cells, and without ART, there is a severe CD4 cell depletion. Microbial translocation from the damaged mucosa, releasing PAMPs (bacterial LPS, 16S rDNA, and CpG DNA) and DAMPs (mtDNA, HMGB1 protein, and defensins), stimulates the production of proinflammatory cytokines (IL-1, IL-6, IL-10, INF-*α*, and TNF-*α*) that promote the activation/inflammatory status, a critical hallmark of HIV infection. Immunodeficiency leads to AIDS-related diseases, including AIDS-defining malignancies. With ART, a small fraction of the virus escapes control and establishes the residual reservoir, which promotes a state of chronic low-grade inflammation/activation, where T and B cell phenotype is altered, with increased expression of senescence markers. Accelerated telomere shortening promotes premature aging and may induce genetic instability. This scenario leads to the development of aging-related illnesses, including non-AIDS-defining malignancies.

In this review, we examine the available data on how HIV and/or ART impact on immune and biological senescence of HIV-infected children. The impact of HIV with and without ART is schematized in Figure 1.

## 2. Clinical Conditions Related to Premature Aging of HIV-Infected Children

Despite the significant improvements due to ART introduction, the life span of HIV-infected children is not yet comparable to that of uninfected ones: their premature aging exposes them to a higher risk of acquiring and developing age-related chronic diseases. The continued release of virions by residual replicating virus (that persists at low levels even in the presence of effective ART) promotes a chronic inflammatory status, in which the release of proinflammatory cytokines favors premature cellular aging and the pathophysiological scenario typically observed in elderly persons. This includes renal and cardiovascular diseases, metabolic and endocrine alterations, cerebrovascular diseases, and malignancies [42–46, 57–59]. Here, we focus on malignancies diagnosed in HIV-infected children.

As happens for HIV-infected adults [60], HIV-infected children show a higher frequency of malignancies compared to the general population [45, 61–65]. In the pre-ART era, the risk of malignancy occurrence was mainly linked to the immunological dysfunction *per se* and lack of adaptive immune response against oncogenic viruses. Kaposi sarcoma (KS) and non-Hodgkin lymphoma (NHL), the two AIDS-defining malignancies (ADM) most frequent in children [61], are indeed associated with Human gammaherpesvirus 8 (HHV8) and Epstein-Barr Virus (EBV), respectively [45, 66–68]. ART introduction led to a change in the nature of HIV-related malignancies, with a reduced incidence of ADM (e.g., KS -87% and NHL -60% [69]) and increased incidence of non-ADM (including Hodgkin's disease, anal cancer, oral squamous carcinoma, hepatocarcinoma, leiomyosarcoma, and Merkel cell carcinoma [45]), especially among immunocompromised HIV-infected children who have received ART for a reduced period of time [61, 70]. The decrease in the incidence of ADM may be attributable to recovery of CD4 cells, partial restoration of immune functions, and lower immune activation, induced by effective ART. As an example, Petrara et al. [71] suggested that limiting HIV replication and related microbial translocation and immune activation may prevent superinfection with EBV or lower EBV viremia, thus reducing the risk of EBV-associated NHL. ART, however, can only partially revert the increased expression of all factors leading to chronic immune activation [72], due to the persistence of residual viremia. Chronic immune activation, with increased cell turnover and premature immune senescence, is indeed associated with the increased risk of non-ADM malignancies in HIV-infected children [17, 67, 73]. The genetic instability conferred by accelerated telomere erosion and the hampered immune surveillance may promote cancer development [74]. Moreover, senescent cells furtherly fuel tumor growth by secretion of inflammatory cytokines, growth factors, and proteases [75, 76], establishing a tumorigenic microenvironment.

## 3. Premature Immune Senescence

The main target of HIV virus is immune cells, in particular CD4 lymphocytes, monocytes, and macrophages [77]. HIV infection leads indeed to a severe depletion of CD4 cells and a progressive loss of function of the innate and adaptive immune system [78]. Despite ART effectiveness, residual HIV infection still has consequences on the immune phenotype of the host: HIV-driven immune senescence is indeed one of the leading contributors to the premature aging displayed by HIV patients [79, 80]. A senescent immune system, characterized by the accumulation of functionally impaired differentiated immune cells, compromises the immune response [81], hampering the ability to react to novel antigen challenges and contributing to frailty [82].

One of the main peculiar features of the children immune system, compared to that of the adults, is their much higher thymic output [23] that constitutes an advantage over adults in the context of HIV infection. On the other hand, the immune system of children exposed to HIV has not yet fully developed when they meet the virus, therefore, leaving them unable to mount an efficient immune response; therefore, the consequences of HIV in this case are more complex and the disease has a faster progression [29]. Moreover, children are exposed to the virus from an early age, in most cases from birth: despite the benefits of an effective therapy, the lifelong exposure to the virus and to the drugs promotes a chronic activation of the immune system, contributing to its premature aging. The main alterations on the immune phenotype of HIV-infected children are described in detail below.

### 3.1. T Cell Compartment

Many alterations in the CD4 and CD8 cell subsets have been reported for all HIV-infected individuals and in particular for children and infants [83–86]. The main alteration in the immune phenotype is the inversion of the CD4/CD8 ratio, which is considered a hallmark of disease progression: whereas a normal CD4/CD8 ratio is above 1, untreated individuals with HIV undergo CD4 depletion, which results in a CD4/CD8 ratio below 1. Several children and adults on ART, despite reaching virological suppression, may not recover to a normal CD4/CD8 ratio, an effect attributable both to incomplete recovery of CD4 and to the increase of CD8 cells due to persistent immune activation. About 66% of HIV-infected children, however, succeed to recover to a normal CD4/CD8 ratio [87], a percentage higher than the one observed in adults [88]; the 33% of children who do not recover are usually older and/or have started ART later. This difference in the ability to restore a normal CD4/CD8 ratio has been partially explained with the increased expansion of T regulatory (Treg) cells of children compared to adults and by the better proliferative capacity of their HIV-specific T cells [89]. Conversely, a study [90] showed that defective recovery of the CD4/CD8 ratio is instead associated to increased levels of activated, senescent and effector memory T cells, with decreased naive T cells. Overall, the whole CD4 and CD8 T cell populations are affected by HIV, even in individuals on effective ART [59].

Naïve T cells undergo a drastic reduction due to thymus involution [91] and to frequent stimulation and expansion of preexisting populations of antigen-specific T cells in the struggle to regenerate the T cell pool [81]. Several studies [84, 92–94] pointed out, indeed, a loss of naïve CD4 and CD8 (CD45RA+CCR7+) cells both in adults and in children; at the same time, a decrease of central memory in favor to the effector memory (CD45RA-CCR7-), with the expansion of CD27-, marker of effector type T cells [95], was detected. Another study [36] compared HIV-infected, HIV-exposed uninfected (HEU), and HIV-unexposed uninfected (HUU) infants, showing that, over the first year of life, CD8 naïve, memory, effector, terminally differentiated, and senescent T cells were significantly altered in HIV-infected infants compared to the other two groups; in particular, CD8 naïve cells were significantly lower, while CD8 effector memory, terminally differentiated (CD45RA+CCR7-), and senescent (CD28-CD57+) cells were significantly higher. A study on 57 perinatally HIV-infected adolescents [39] showed increased levels of senescence and proliferation (Ki67+) markers in the memory CD4 cell subset, compared to healthy subjects; their effector memory cells were also positive for activation marker HLA-DR. A recent study [56] compared 71 HIV-infected children below 5 years of age to HEU and HUU cohorts, observing an accelerated senescence of both their CD4 and CD8 cell compartments, with significantly higher percentages of activated (CD38+HLA-DR+) and exhausted (programmed cell death, PD-1+) CD4 cells and of activated, senescent, and exhausted CD8 cells; interestingly, the exposure to ART prophylaxis of HEU children did not negatively affect their immune phenotype.

Other studies confirmed that HIV-infected children display higher percentages of exhausted T cells, which often fail to recover despite treatment [96, 97]; PD-1 expression, the principal marker of HIV-related cell exhaustion, has indeed been proposed as a disease progression marker [97]. Recently, additional immune checkpoint inhibitors (ICIs), including CTLA-4, TIM3, LAG3, TIGIT, 2B4, and CD160, were identified; they were found to coexpress especially in viremic progressors, furtherly inhibiting T cell function [98]. In spite of the promising potential of ICIs as progression markers, exploring their expression in HIV-infected children is still an open field of research.

Treg (CD4+CD25+CD127-FoxP3+) cells limit excessive or inappropriate immune activation against antigens, and in particular, they prevent responsiveness to self-antigens [99–101]. In the HIV-infection context, different studies highlight two opposite effects of Tregs; they are suggested not only to decrease excessive immune activation [102–104] but, on the other hand, also to suppress HIV-specific immune response [105–108]. A study on 6-14-year-old HIV-infected children [109] showed that viremia significantly correlates with the percentage of Tregs; higher percentage of Tregs is also associated with higher immune activation and higher HIV-DNA levels, suggesting that the regulatory function of this subset does not suffice to limit immune activation.

T follicular helper (Tfh) cells are specialized CD4 cell subset whose signaling function allows the generation of long-lived B cells during the immune response. Indeed, they are considered a biomarker of vaccine response. The equilibrium between their different subsets (i.e., Th1, Th2, and Th17) and their function is perturbed during HIV infection, even under virological control, resulting in an impaired response to vaccination. In particular, in HIV-infected children, a negative response to vaccination has been associated with Tfh cells coexpressing multiple activation markers [110, 111].

Increased immune senescent phenotype in HIV-infected children has been pointed out by many studies [38, 112, 113]. Moreover, the persistent immune activation and exhaustion, together with alterations of memory T cells, may also have an impact on the efficacy of childhood vaccination and have been linked to poor response to vaccines and higher risk to acquire vaccine-preventable diseases [36, 111–117].

### 3.2. B Cell Compartment

The B cell compartment is impacted by similar alterations as those affecting T cells: indeed, several studies [114, 118, 119] demonstrated that HIV-infected children, even with undetectable viral load, show B cell alterations typical of older healthy controls, such as an increased number of mature-activated (CD10- CD21-) and senescent double-negative (IgD- CD27-) B cells. Other B cell alterations linked with chronic HIV persistence include increased percentages of immature transitional (CD10+/++ CD21low/high CD27-), activated memory (CD10- CD21low CD27+), and exhausted memory (CD10- CD21low CD27-) B cells and decreased percentages of resting memory (CD10- CD21high CD27+) B cell subset [120, 121]. A study [122] on ART-naïve children below 2 years of age revealed many alterations of their B cell phenotype compared to the uninfected control group: they had significant depletion of naïve (IgD+ CD27-), nonswitched memory (NSM, IgD+ CD27+), naïve mature (CD21high CD27-), and activated (CD25+) B cells and significant expansion of double negative, activated memory (CD21low CD27+), tissue-like memory (TLM, CD21low CD27-), and apoptosis-prone (CD95+) B cells. ART-naïve children suffered a progressive deterioration over 1-year follow-up, with further depletion of naïve and NSM cells and expansion of double-negative B cell subset. On the other hand, one year of ART could only partially restore these alterations: there was an increase in the naïve, NSM, and naïve mature cell subsets and a decrease in the double-negative, activated memory, and TLM subsets; however, no improvement was found in the resting memory, activated, and apoptosis-prone B cell subsets that remained significantly altered. Thus, as for T cell compartment, ART does not fully restore B cell functionality. Notably, despite ART, homing of B cells to germinal center is defected with the consequent impaired vaccine responses in HIV-infected children [114, 123, 124]. In addition, the expression of B cell genes, including those involved in the inflammation and aging, that could predict the response to vaccination [125, 126], remains perturbed even after a stable and long virological control.

## 4. Chronic Immune Activation and Persistent Inflammation

Persistent inflammation and chronic immune activation are leading causes of the senescent pathway that favors the risk of non-AIDS morbidity and mortality in HIV-infected children. T cell activation, marked by CD38 and HLA-DR coexpression on CD8 T cells, is a prognostic indicator for disease progression at different stages of HIV infection [127]. Moreover, HIV infection drives the microbial translocation [128]: the massive depletion of CD4 cells associated with HIV infection induces an impairment of mucosal surface integrity in the gut and leads to the release of pathogen-associated molecular patterns (PAMPs, such as bacterial lipopolysaccharide, 16S ribosomal DNA, and CpG DNA [55]) and damage-associated molecular patterns (DAMPs, such as mitochondrial DNA, high-mobility group box 1 protein, and defensins [129, 130]) into the circulation. PAMPs and DAMPs activate the immune system by binding to the extra- or intracellular domain of Toll-like receptors (TLRs), which are involved in the host inflammatory response, initiating a complex-signal transduction cascade which, via the NF-*κ*B pathway [131], ultimately leads to increased transcription of proinflammatory cytokines (such as IL-6, IL-10, and interferon-*α*) that may play a role in establishing a protumorigenic inflammatory environment [66].

Several studies [132–134] have suggested that, despite viral suppression, children with perinatally acquired HIV have higher levels of inflammation, immune activation, and alterations in intestinal permeability, compared to HEU and HUU children. Notably, immune activation is higher in viremic than aviremic children, but microbial translocation may occur regardless of viremia and T cell activation. While ART in HIV-infected subjects generally allows for immune reconstitution in peripheral blood, reconstitution of the gastrointestinal tract occurs at a much slower pace, and both immunological and structural abnormalities persist in the gastrointestinal tract, thus explaining the residual inflammation and heightened morbidities in HIV-infected ART recipients [135]. In a cohort of HIV-infected children [136], ART initiation rapidly and persistently reversed T cell activation but failed to normalize CD4/CD8 ratios and plasma sCD14 levels. However, another study on a cohort of perinatally HIV-infected children [137] showed that ART initiation normalized sCD163 (marker of monocyte activation) levels and improved long-term pediatric outcomes. A recent study [138] agreed that immune activation decreases over time in children after starting ART, which does not have adverse effects itself on microbial translocation.

To support the concept that persistent immune activation and cellular exhaustion are closely linked to accelerated biological aging and immune senescence, Gianesin et al. [56] found that HIV-infected children accumulate activated and exhausted CD8 T cells together with a higher percentage of senescent CD8 T cells, which are all inversely correlated with telomere length. The immune exhaustion is also increased in HIV-infected individuals despite viral suppression [139]. Indeed, PD-1 is the eligible marker of immune exhaustion of T cells, and its increased expression levels predict the rate of HIV disease progression in adults [140, 141]. HIV-infected children have increased PD-1 expression on CD8 T cells that correlates with immune activation [142, 143]. It was recently demonstrated that CD4 cells expressing PD-1 constitute an important source of persistent viral replication in ART-treated individuals, and the contribution of PD-1+ CD4 cells to the persistent reservoir progressively decreased with increased length of ART [144].

Immune activation also results in chronic stimulation and expansion of B cells. ART allows, at least partially, the normalization of activated B cell subsets and age-dependent accumulation of resting memory B cells [145]. However, as for T cell, ART does not eliminate B cell activation. In HIV-infected adults, immune activation persists over time and is susceptible to therapy; indeed, compared to classical combined ART, a monotherapy with protease inhibitors has a lower control on DAMP levels and B cell hyperactivation, so it may have lower control on EBV reactivation and/or polyclonal expansion of EBV-infected B cells and, thus, on the onset of EBV-related malignancies [146]. Few data are available about B cell activation in HIV-infected children. A study [71] demonstrated that children on ART have significant lower levels of microbial translocation and EBV levels than ART-naïve children. Recently, a study [147] showed that pre-ART progressors had higher percentages of mature activated and TLM cells and higher plasma levels of IL-4, IL-6, IL-10, and IgA compared to seronegative controls. After ART initiation, levels of proinflammatory cytokines IL-4, IL10, and IgG significantly lowered.

Overall, all these reports agree that, despite ART, microbial translocation persists and leads to a chronic low-grade inflammation. In HIV-infected children, the monitoring of persistent inflammation/immune activation and immune exhaustion will be of clinical importance to estimate the rate of premature aging and its associated production of inflammatory cytokines, as pivotal factors acting in the pathogenesis of premature aging and malignancies.

## 5. Premature Biological Aging

Telomeres are involved in cellular aging and immune senescence mechanisms. Telomeres are long tandem repeated DNA sequences (TTAGGG) at the end of chromosomes that are essential for protection of chromosome integrity, preventing end-to-end fusion and DNA degradation [148]. The ribonucleoprotein complex telomerase has the function of maintaining telomeres by synthesizing new telomeric repeats; its activity is usually not detected in somatic cells due to the downregulation of its catalytic protein TERT, which is instead expressed during embryogenesis, in rapidly dividing tissues and in the vast majority of tumors [148]. During each cell division, DNA polymerase is unable to copy the end of chromosomes (the end-replication problem); thus, some of the telomere repeats are lost. After several cell divisions, telomere length reaches a critical threshold, below which cells stop dividing and physiologically undergo senescence or trigger genomic instability, that may promote age-associated diseases and tumor development [149].

Telomeres get naturally shorter with age [150]; telomere length is therefore a valid biomarker of aging in the general population, and accelerated telomere shortening leads to premature aging, which is correlated with several pathologies [151, 152]. The causes leading to accelerated telomere shortening can be, however, diverse. Indeed, telomere length and their shortening rate are not only associated with genetic factors, gender, and ethnicity, but they are also influenced by different behavioral and environmental factors, such as stress, physical activity, dietary habits, smoke, and alcohol consumption.

### 5.1. Telomere Implications in Diseases

Telomere length is associated with age-related diseases and decreased life span. Several studies linked shorter telomeres and telomere attrition with increased risk and increased severity of cardiovascular diseases, stroke, heart attack, and mortality [153–159]. As an example, a study on elderly patients [153] showed that, among the 143 studied patients, the 71 with shortest telomeres had a 3- and 8-fold higher mortality rate due to heart and infectious diseases, respectively. Premature aging disorders, among which progeria, Nijmegen breakage, Cockayne and Down syndromes, and dyskeratosis congenita are associated with shorter telomeres; instead, others like Werner and Boom syndromes and Ataxia telangiectasia are associated with an accelerated telomere shortening [160]. In contrast to the clear association of the aforementioned aging conditions with telomere length or erosion, similar studies on other conditions raised conflicting results: it is not yet fully cleared whether type II diabetes [161, 162] and Alzheimer's [163–167] and Parkinson's [168, 169] diseases are associated with telomere shortening or if having shorter telomeres is a risk factor for such conditions. Moreover, telomere dynamics is intrinsically related with the tumorigenesis mechanism. Indeed, shortening of telomeres below a critical level triggers the pathways that lead to cell senescence, when genomic instability is increased [170]. Should the apoptotic mechanism fail, cells may acquire immortality (mainly through the upregulation of telomerase) and, thus, tumorigenesis mechanisms may begin. Telomeres have therefore the potential to be both beneficial and detrimental factors, whether they are recognized in the signaling pathway resulting in cell apoptosis or not. In agreement with this dual role, some types of cancers have been associated with shortened telomeres while others with elongated telomeres [171].

Telomeres are not only involved in diseases affecting the elderly population, but they also have a role in conditions affecting children and adolescents. A recent study [172] on 62 children and adolescents diagnosed with AATD (*α*_1_-antitrypsin deficiency) and with intermediate to high risk for developing lung or liver damage showed that they had significantly shorter telomeres and increased oxidative stress than controls; high-risk patients showed not only shorter telomeres but also lower TERT expression and decreased telomerase activity than the other groups. Another study [173] on 44 patients, among which 26 children, with inherited telomere biology disorders (such as dyskeratosis congenita, Hoyeraal-Hreidarsson, and Revesz syndrome) showed that 57% of them had at least one structural brain abnormality or variant; they also had psychiatric diagnoses and other diseases more frequently than the general population. Another confirmation comes from a study [174] conducted on 47 young adults (17-24 years old) diagnosed with the premature aging syndrome of Prader-Willi (PWS). They displayed significantly shorter telomere length compared to age-matched healthy controls; they also showed a mild association with lower IQ. Childhood cancers sometimes need to be addressed differently from adult ones; one of the reasons resides in the differences in the mutational landscapes and the prevalence of telomere maintenance mechanisms [175]. Environmental and behavioral factors might also impact on telomere length and erosion in newborns, children, and adolescents. For example, a study on 762 mother-newborn pairs in China [176] demonstrated that prenatal exposure to some phthalate metabolites was associated with shorter cord blood telomere length; this study was also an evidence that intrauterine environment has the potential to impact newborns' telomere length. Different studies on European cohorts [177, 178] showed that higher child adiposity indicators are associated with short telomeres in children; overweight and obesity in childhood and adolescence are associated with shorter telomeres; therefore, an increased BMI early in life may be associated with accelerated biological aging and may have an adverse impact on future health during adulthood.

To summarize, telomere length has the potential to have a valid diagnostic significance in specific settings. A recent work by Alder et al. [179] measured telomeres of 100 individuals with known pathogenic mutations in telomerase and other telomere maintenance genes, compared with those of 636 healthy individuals of all ages. All of the 100 patients had age-adjusted telomere length below the 50^th^ percentile: this indicated a 100% negative predictive value for identifying a clinically relevant mutation in telomerase/telomere maintenance genes. Moreover, a significant correlation was found between faster telomere attrition and earlier onset of idiopathic bone marrow failure; 25% of the idiopathic bone marrow failure patients had their treatment regimen choice modified based on their telomere length measurement, resulting in an improvement of their clinical outcomes.

### 5.2. Telomere Shortening in HIV-Infected Children and Impact of ART

There is nowadays evidence that HIV-infected individuals have overall shorter telomeres than uninfected controls [180–183], implicating that HIV directly influences telomere attrition, which occurs early after infection [184]. It has also been suggested that antiretroviral drugs themselves can impact on accelerated aging. Indeed, HIV reverse transcriptase shares homology with TERT [148, 185, 186]; therefore, nucleoside reverse transcriptase inhibitors (NRTIs), such as zidovudine (ZDV) or abacavir, might also inhibit TERT. In vitro studies showed that NRTIs inhibit telomerase causing an accelerated erosion of telomeres [53, 54, 187, 188]; recent studies argued that the effect of prophylaxis and therapy is negligible compared to that of the infection itself [56]. However, two studies published in 2018 still reported apparently conflicting results on the impact of different NRTI regimens on patients' telomere length and their change over time [189, 190]. The main findings of the papers presented in this paragraph are also summarized in Table 1.

A study [37] on 94 0-19-year-old HIV-infected children found no significant differences in their relative telomere length compared to that of exposed (HEU) and unexposed (HUU) uninfected controls. However, in the HIV-positive group, higher viral load was associated with shortening of telomeres. To investigate the impact of NRTIs on children's telomeres, a study was conducted on 114 HEU infants exposed to ZDV prophylaxis [191]; their telomeres at birth were similar to those of HUU controls, and no association was found between telomere length and maternal ART regimen. Among the 114 HEU children, those exposed to maternal ZDV+lamivudine+nelfinavir/nevirapine regimen had longer telomeres at birth. Moreover, telomere attrition in HEU children was more rapid in their first year of life compared to that in HUU children, but then it normalized. In agreement with these findings, a recent study [192] on 94 HEU ZDV-exposed and 85 HEU ZDV-unexposed newborns revealed that telomere length of the ZDV-exposed infants was longer compared to that of ZDV-unexposed ones. This study also found a correlation between high maternal plasma viremia levels and shorter infants' telomeres. In partial conflict with these evidences, a recent study [193] on 120 HIV-infected children below 6 years of age who started ART before 2 years of age found that telomeres of HUU children were significantly longer than those of HIV-infected and HEU children; instead, HIV-infected and HEU children had similar telomere lengths. In addition, this study did not find any relationship between telomere length and markers of inflammation (IL-6, TNF-*α*, high-sensitivity CRP, and sCD14). Only one study [56] analyzed both the immune senescence profile and telomere length in 0-5-year-old HIV-infected, HEU, and HUU children. In this study, telomeres were significantly shorter in HIV-infected children compared to HEU and HUU ones; moreover, among the HIV-infected children, telomeres were shorter in ART-naïve than in ART-treated children. HIV-infected children also displayed significantly higher percentages of senescent (CD28- CD57+), activated (CD38+ HLADR+), and exhausted (PD1+) CD8 T cells, while these percentages were comparable between HEU and HUU children. The inverse correlation found between activated, exhausted, and senescent CD8 cells and telomere length corroborated the idea that persistent immune activation is closely linked to accelerated biological aging and immune senescence. Given the need of maintaining children with HIV on ART for their entire life span, it is of interest to investigate the long-term consequences of perinatally acquired HIV. A 2019 study [194] investigated telomere length and erosion, together with thymic and bone marrow output, of young adults (median age 27 years old) who acquired HIV perinatally compared to age-matched individuals infected later in life. Both groups showed a normal thymic output and normal CD4 count; however, both groups had shorter telomeres and a faster telomere erosion compared to uninfected age-matched controls. This apparent discrepancy has been explained proposing that the attempt to control the infection continuously recruits naïve cells, which shift to the memory phenotype. Moreover, the positive correlation that was found between CD4 count and telomere length in both HIV-infected groups furtherly supports the concept that CD4 cells are newly recruited cells which underwent fewer cell divisions.

The collection of these data supports the idea that HIV infection itself has the major detrimental impact on cellular aging, and ART benefits strongly outweigh the negative effects on telomeres.

## 6. Conclusions

ART has changed the natural history of HIV, which is now considerably a chronic disease.

Despite effectiveness of treatments, HIV-infected children still do not have the same life expectancy and quality of life compared to the general population. Perinatally infected children acquire the virus early, when their immune system has not yet reached full development and they are unable to mount efficient immune response, so the disease has a faster progression. A persistent state of inflammation and activation of the immune system contributes to establishing a premature aging profile. This cannot be fully reverted by ART, representing one of the main causes of comorbidities/malignancies in treated HIV-infected children. Accumulating evidence demonstrated that the beneficial effects of ART greatly outweigh the potential side effects of NRTI use; indeed, it is mainly HIV that induces telomere attrition and premature aging. In this setting, the monitoring of markers of inflammation/immune activation and premature aging is of great clinical relevance. Early-treated children with reduced inflammatory and senescent status could reveal optimal candidates for future treatments and vaccine trials.

## Figures and Tables

**Table 1:** Summary of recent findings on telomeres on HIV-infected children, adolescents, and young adults.

Patients	Median age [IQR] (y.o.)	Telomere length median [IQR]	Method of telomere measure	Main findings	Authors and reference
94 pHIV	13.3 [9.9-15.8]	n.d.	Relative TL by rtPCR	Telomere attrition is similar for pHIV, HEU, and HUU. Older age and male gender are correlated with shorter TL. Detectable viremia and absence of ART are correlated with shorter TL.	Cote et al. [37]
177 HEU	1.7 [0.6-4.0]	n.d.			
104 HUU	10.6 [5.3-14.2]	n.d.			
71 pHIV	3.11 [1.40-4.48]	2.21 [1.94-2.58]	Relative TL by rtPCR	pHIV have significantly shorter TL than HEU and HUU. ART-naïve pHIV have shorter TL than pHIV on ART. Percentages of senescent, activated, and exhausted CD8 cells are higher in pHIV than in HEU and HUU.	Gianesin et al. [56]
65 HEU	1.74 [0.99-3.31]	2.63 [2.25-3.21]			
56 HUU	1.85 [0.84-3.46]	2.88 [2.49-3.10]			
324 HEU	≥1 samples 0-3 y.o.	n.d.	Relative TL by rtPCR	TL is similar between HEU and HUU. HIV and cART exposure in utero does not appear to alter telomere dynamics during early life.	Ajaykumar et al. [191]
306 HUU	1 sample 0-3 y.o.	n.d.			
120 pHIV	6.4 ± 1.4	4.14 ± 0.85	Absolute TL by rtPCR	There was no evidence of accelerated biological aging by mDNA levels. Absolute telomere length was shorter in pHIV and HEU compared to HUU but did not differ between pHIV and HEU.	Shiau et al. [193]
33 HEU	6.1 ± 1.5	4.05 ± 0.74			
25 HUU	6.9 ± 1.1	4.53 ± 0.79			
94 HEU ZDV+	1.0 [0.0-7.0] days	0.85 ± 0.23	Relative TL by rtPCR	TL of ZDV+HEU infants is longer compared to that of ZDV-HEU.	Wang et al. [192]
85 HEU ZDV-	1.0 [0.0-6.9] days	0.65 ± 0.19			
21 pHIV	27 [24-29]	1.0 [0.8-1.2]^∗^	Relative TL by rtPCR	TL and telomere shortening rate of pHIV and npHIV is significantly lower than that of HUU. pHIV and npHIV maintain a normal thymic output, with a continuous shift of the naïve pool into memory subsets. This phenomenon may allow to control viral infection and maintain peripheral homeostasis.	Paghera et al. [194]
19 npHIV	27 [24-29]	0.9 [0.7-1.2]^∗^			
40 HUU	28 [24-31]	1.5 [1.3-1.9]^∗^			

pHIV: perinatally HIV-infected children; npHIV: nonperinatally HIV-infected children; HEU: HIV-exposed uninfected children; HUU: HIV-unexposed uninfected children; HUU: HIV unexposed uninfected; ZDV: zidovudine; TL: telomere length; n.d.: values not reported in the original papers; ^∗^values estimated from Figure 2A of Paghera et al. [194].
